# Using Parameter Constraints to Choose State Structures in Cost-Effectiveness Modelling

**DOI:** 10.1007/s40273-017-0501-9

**Published:** 2017-03-24

**Authors:** Howard Thom, Chris Jackson, Nicky Welton, Linda Sharples

**Affiliations:** 1 School of Social and Community Medicine, University of Bristol, Bristol, UK; 2 Medical Research Council Biostatistics Unit, Cambridge, UK; 3 Department of Medical Statistics, London School of Hygiene and Tropical Medicine, London, UK

## Abstract

**Background:**

This article addresses the choice of state structure in a cost-effectiveness multi-state model. Key model outputs, such as treatment recommendations and prioritisation of future research, may be sensitive to state structure choice. For example, it may be uncertain whether to consider similar disease severities or similar clinical events as the same state or as separate states. Standard statistical methods for comparing models require a common reference dataset but merging states in a model aggregates the data, rendering these methods invalid.

**Methods:**

We propose a method that involves re-expressing a model with merged states as a model on the larger state space in which particular transition probabilities, costs and utilities are constrained to be equal between states. This produces a model that gives identical estimates of cost effectiveness to the model with merged states, while leaving the data unchanged. The comparison of state structures can be achieved by comparing maximised likelihoods or information criteria between constrained and unconstrained models. We can thus test whether the costs and/or health consequences for a patient in two states are the same, and hence if the states can be merged. We note that different structures can be used for rates, costs and utilities, as appropriate.

**Application:**

We illustrate our method with applications to two recent models evaluating the cost effectiveness of prescribing anti-depressant medications by depression severity and the cost effectiveness of diagnostic tests for coronary artery disease.

**Conclusions:**

State structures in cost-effectiveness models can be compared using standard methods to compare constrained and unconstrained models.

**Electronic supplementary material:**

The online version of this article (doi:10.1007/s40273-017-0501-9) contains supplementary material, which is available to authorized users.

## Key Points for Decision Makers

**Table:** 

State-transition cost-effectiveness models with different state structures can give different recommendations on treatment decisions or research prioritisation. To date, there have been no formal statistical methods described for comparing different state structures.
Merging two states in a transition model, such as similar types of event, is practically equivalent to constraining the outward transition probabilities, costs and utilities to be equal for the two states. Thus, the state structures can be compared by assessing whether these constraints are reasonable. This can be done using standard methods for comparing statistical models, and suitable data.
For example, comparing transition probabilities requires data consisting of the numbers of patients observed to transition out of the states of interest to each potential destination. To compare costs and utilities between states, individual-level samples are required. Maximum likelihood and Akaike’s information criterion can then be used to assess the constraints. If such data are not available, they might be derived from published summaries, or the comparison can be made informally.

## Introduction

Health economic evaluations rely on cost-effectiveness models, such as Markov multi-state models [1], to produce accurate comparative assessments of the costs and health effects of different interventions for the management of disease. Given a cost-effectiveness model, there may be uncertainty about the correct transition probabilities, costs or utilities. This is commonly termed parameter uncertainty and managed by probabilistic sensitivity analysis [2, 3]. Research recommendations can also be guided by the expected value of perfect information (EVPI) and expected value of partial perfect information, comparing the benefits in terms of costs and monetised health effects gained from a decision based on evidence, where parameter uncertainty is removed or reduced, with that based on current evidence [4].

**Fig. 1:**
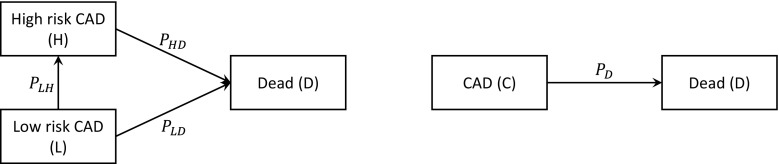
Coronary artery disease (CAD) models with split and merged CAD severity. *P*_XY_ is the probability of making a transition from state X to state Y in a cycle

However, all models are idealised representations and the choice of structure for the model may be uncertain. Moreover, different choices can change decision recommendations, as found in models for breast cancer and in varicella vaccination [5, 6]. In this article, we consider uncertainty about the choice of states in a state-transition health economic model, a subject which has, to our knowledge, not yet been formally addressed. An example in coronary artery disease (CAD) is the choice between a model with split and merged severities of CAD, illustrated in Fig. 1. The split-state model divides the ‘CAD’ state into ‘high-risk CAD’ and ‘low-risk CAD’, as severity may have an effect on costs, health utilities and the probability of death. These structural choices are currently made informally, on the basis of clinical opinion and the availability of data [7]. Guidelines recommend scenario analyses or parameterising structural uncertainties [2, 8, 9], but it is often unclear how they can be parameterised.

Formal statistical approaches for comparing model structures against the data used to build them include the Akaike information criterion (AIC) [10, 11] and, for Bayesian models, the deviance information criterion [12, 13]. These trade off the fit to the data, represented by the likelihood, with the complexity, related to the effective number of parameters, of the models. Thus, they can find the optimal balance between the risk of bias (from excluding important events or predictors) and the reduced uncertainty in the estimates from a smaller model. These criteria can also be used to construct weighted averages over the possible structures [11, 14]. However, these methods are only valid for models fit to the same datasets and it has been shown that multi-state models with different state structures use different datasets [15]. Another approach is to split the model into a series of sub-functions and add discrepancy parameters to the outputs of these functions to represent state structure uncertainty [16]. However, the discrepancies do not indicate which assumptions are more plausible and can be difficult to interpret for complex models. A further approach is to compare the ability of the models to predict the events represented by both models [15, 17], in the above CAD example, this would be CAD and death. However, calculating the appropriate measure of fit for the restricted information criteria described in this article is technically demanding. State structures might also be compared by informal validation against external data if available.

We propose a method that allows the choice between state structures to be parameterised, and for which standard likelihood-based model selection criteria are valid. This enables us to compare structures under the principle that similar states may be merged if the consequences of occupying them are the same. Here, the ‘consequences’ for a patient consist of the potential exit states, the probabilities of transition to these exit states, the costs and the utilities. We show that smaller models can be reformulated into practically equivalent models on the larger state space by constraining the outward transition probabilities, costs and utilities to be the same for the ‘merged’ states. The model choice is then a matter of assessing whether each of these constraints is reasonable, based on the fit to data. We also consider using ‘partially merged’ models with different state structures for transition probabilities, costs and utilities, depending on the most appropriate choice for each consequence; for example, we may assume the costs in high-risk CAD ($c_{\text{H}}$) to be the same as those in low-risk CAD ($c_{\text{L}}$) but that the transition probabilities to the dead state ($P_{\text{HD}}$ and $P_{\text{LD}}$) are different. We illustrate our approach in models comparing treatment strategies for the management of depression, and diagnostic tests for CAD.

## Methods for Comparing State Structures by Assessing Parameter Constraints

Suppose we have data consisting of the numbers of individuals who are observed to make a transition between each pair of states over a particular time interval, and corresponding denominators of the total number of patients at risk. The models are fitted to these data by maximum likelihood or Bayesian estimation, giving estimates of transition probabilities between states over one cycle of a discrete-time model [18]. If such data are not explicitly available, they might be derived from related data (such as published relative risks of death) under weak assumptions, and we discuss an example in Sect. 4. Costs and utilities for states are estimated from samples of individual-level costs and utilities, or from published unit costs combined with assumptions, expert beliefs or data on individual resource use.

### Merging Two States with One Common Exit State

Consider again the split- and merged-state models for CAD presented in Fig. 1. It is intuitive that if we impose the constraint:

1 $$P_{\text{HD}} = P_{\text{LD}} = P_{\text{D}} ,$$

the fitted models should give the same predictions of expected survival. We prove this formally in Appendix 1 in ESM by showing that the likelihood of the split-state model, subject to the above constraint, is proportional to that of the merged-state model, with a proportionality factor that is independent of $P_{\text{D}}$. Thus, the estimate of $P_{\text{D}}$, and thus the expected survival over any time horizon, will be identical under both the constrained split- and merged-state models. This also applies to Bayesian estimation if the prior on $P_{\text{D}}$ is the same in the merged and constrained models, and to ‘reversible’ models where the transition back from high to low is permitted because the probability of death $P_{\text{D}}$would remain independent of the disease state. Furthermore, if we also constrain the costs and utilities of the states to be equal, as $c_{\text{H}} = c_{\text{L}} = c$ and $u_{\text{H}} = u_{\text{L}} = u$, the models will give the same predictions of lifetime costs and quality-adjusted survival, and hence the same decision recommendations.

Thus, the uncertainty regarding state structure has been parameterised, as a choice of whether these three constraints are reasonable. If all three are supported by the data, the merged model can be used because it is equivalent to the constrained split model. If all constraints are invalid, then the fully split model is most appropriate. A ‘partially merged’ model can also be recommended, for example, if the transition probabilities but not the costs are found to be equivalent.

### Merging Any Number of States with Any Number of Exit States

**Fig. 2:**
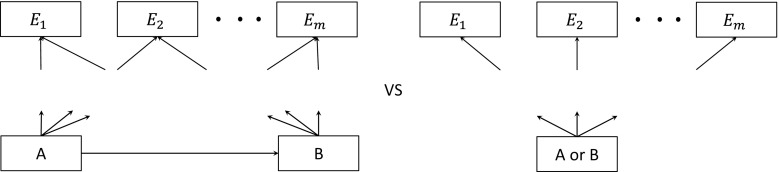
Merging states with any number of exit states. States A and B are the states under consideration for merging while *E*_*i*_ is a set of arbitrary exit states

The principle and procedure outlined above apply to models in which the states to be merged have any number of ‘exit states’, for example, different causes of death, provided the exit states are common to the states to be merged. Figure 2 illustrates two models; one splits states A and B while the second merges these states. The exit states, $E_{1} , \ldots ,E_{m}$, are the same for states A and B. To make the split-state model equivalent to the merged state-model, we use the constraints

$$P_{{AE_{i} }} = P_{{BE_{i} }} ,$$

for $i = 1, \ldots , m$, where $P_{{AE_{i} }}$ and $P_{{BE_{i} }}$ are the probability of transiting to state $E_{i}$ from A and B, respectively, and constrain the costs and utilities as before, $c_{A} = c_{B} = c$ and $u_{A} = u_{B} = u$. Thus, the model choice involves determining, for each *i,* whether the probability of death from cause *i*, the cost and the utility depends on the disease status being A or B.

**Fig. 3:**
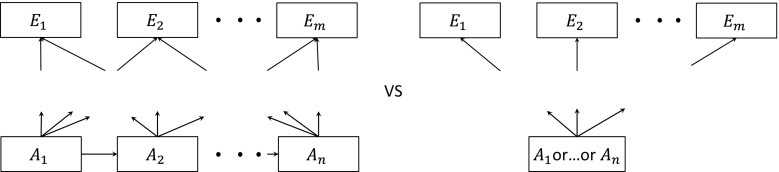
Merging any number of states with any number of exit transitions. States *A*_*j*_ are the states under consideration for merging and *E*_*i*_ is a set of arbitrary exit states

A further generalisation is illustrated in Fig. 3. In this case, we consider merging *n* states $A_{1} , \ldots ,A_{n}$ with transitions to *m* states $E_{1} , \ldots ,E_{m}$. The necessary constraints are

2 $$P_{{A_{1} E_{i} }} = P_{{A_{2} E_{i} }} = \cdots = P_{{A_{n} E_{i} }} ,$$

for $i = 1, \ldots , m$, and again $c_{{A_{j} }} = c {\text{ and }} u_{{A_{j} }} = u , {\text{ for }} j = 1, \ldots , n.$ The model can be fully reversible and any transitions can be allowed between the merging states *A*_*j*_. The constrained likelihood of the split-state model is proportional to that of the merged-state model, as proven formally in Appendix 2 in ESM.

The states *A*_*j*_ may represent severities of disease, and the *E*_*i*_ different causes of death, but this result is entirely general to problems of whether to split or combine a set of states $A_{1} , \ldots ,A_{n}$ for which the potential destination states after leaving the set are the same for each $i = 1, \ldots , n$. The ‘split’ and ‘merged’ models shown in Fig. 3 may both be part of a common larger state structure, for example, there may also be transitions into the *A*_*j*_, or into and out of the *E*_*i*_. However, only the constraints (2) on the outward transition probabilities, costs and utilities are required to effectively ‘merge’ the states. Thus, the choice of structures is parameterised as a choice of whether the outward transition probabilities, costs and utilities are common between $A_{1} , \ldots ,A_{n}$.

### Merging States with Different Exit Transitions

**Fig. 4:**
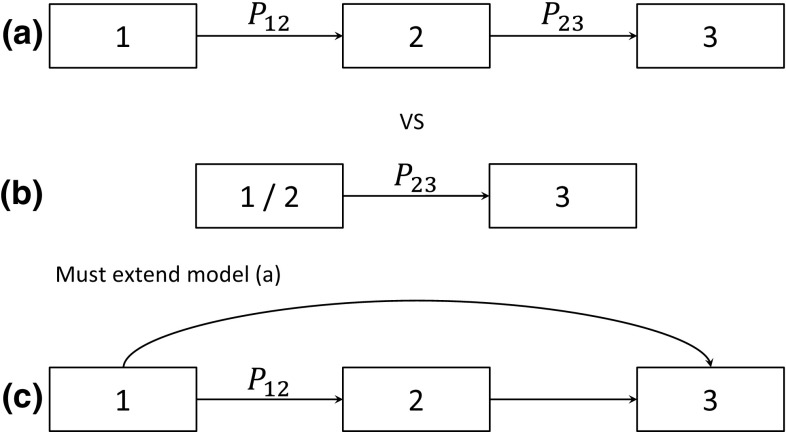
Comparison of two models (**a**) and (**b**) where the states to be merged (1 and 2) have different exit states. These can be compared by comparing constrained versions of the model (**c**), an extended version of model (**a**). *P*_XY_ is the probability of making a transition from state X to state Y in a cycle

An adaptation is required when the states being merged have different exit states, as illustrated by models (a) and (b) in Fig. 4. This is a special case of the structure in Fig. 1, where we know that the probabilities of death are different ($P_{13} = 0$ and $P_{23} \ne 0$) between the states being considered for merging. In discrete time, there is no choice of parameters for which model (a) is equivalent to (b) as a patient in state 2 may exit directly to state 3, but even with $P_{12} = 1$, a patient starting in state 1 would take at least two cycles to reach state 3.

However, we can extend model (**a**) by including a non-zero transition between states 1 and 3 ($P_{13} \ne 0$) to obtain model (c) in Fig. 4. This model can be constrained to model (a) by setting $P_{13} = 0$ or to model (b) by setting $P_{13} = P_{23}$. A comparison between models (a) and (b) is then possible by assessing these constraints on model (c).

## Application to a Markov Model with Individual Patient Data: PANDA

In this section, we present an application to a health economic model for patients with symptoms of depression for whom their general practitioner is considering prescribing anti-depressant medication. The model was used to compare the cost effectiveness of severity thresholds above which to treat patients with depression with anti-depressant medication, and to estimate the value of a proposed randomised controlled trial to compare severity thresholds. The severity of symptoms was measured on the Hamilton Depression Rating (HAMD) scale, and three alternative treatment thresholds (HAMD > 2, HAMD > 15 or HAMD > 25) are compared with a policy of no treatment.

### Model for Cost Effectiveness of Anti-Depressant Treatment by Depression Severity

**Fig. 5:**
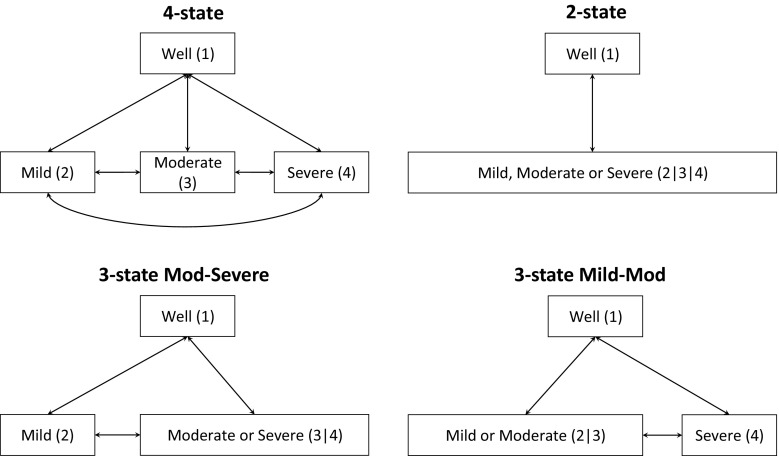
Alternative state structures for the PANDA multi-state depression model. The same structure is assumed for the treated and untreated component of the longer-term PANDA depression model. *PANDA* Prescribing ANtiDepressants that will leAd to a clinical benefit study

The model consists of a short- and a long-term component. The short-term model uses linear regression based on published studies [19–21] to predict a patient’s HAMD score over the first 12 weeks after treatment initiation. The long-term component is a discrete-time Markov multi-state model with a 12-week cycle length and a time horizon of 96 weeks (eight cycles). Patients move between four states of severity: well 0–7 HAMD, mild 8–13 HAMD, moderate 14–18 HAMD and severe/very severe 19–30 HAMD. These are standard categories defined by the American Psychiatric Association [22]. This four-state model is illustrated in Fig. 5. The joint likelihood of the observed data is the product of the probabilities of making the transitions we observed, along with terms for the likelihoods of observed costs and health valuations of observed state occupancies. The transition probabilities are estimated by maximum likelihood from the numbers of individuals observed to move between each pair of states in merged data from the control arms of the IPCRESS, THREshold for AntiDepressant response (THREAD) and TREAD studies [23–25]. Log-normal distributions were used for state costs. These depended on dosing and monitoring regimes inferred from expert clinical opinion and publicly available drug and services costs [26, 27]. As clinical evidence and opinion was that anti-depressant medications have no effect on transition probabilities beyond the initial 12-week period [28], we used the same probabilities between the categories of depression severity in the treated and untreated components. However, the distributions of HAMD at 12 weeks will differ between treated and untreated patients, as will their costs. Owing to a lack of reliable evidence, state utilities were not modelled directly. We instead mapped incremental gains in HAMD, defined as the difference between the mid-points of the category range, to incremental health utilities using published evidence [29–31].

### Alternative Model Structures and Results

The transition probabilities between the four states are informed only by the individual transition history data, and there is no prior clinical belief regarding, for example, how the transition probability to well differs between mild, moderate and severe. Therefore, it is possible that, for these data, a more parsimonious structure that merges two or more of these states could give more precise estimates of cost effectiveness. Thus, we consider a ‘two-state’ model merging all depression states, a ‘Mod-Severe’ model merging the moderate and severe states, and a ‘Mild-Mod’ model merging the mild and moderate states. Merged IPCRESS, THREAD and TREAD data were re-analysed to estimate these transition probabilities for each structure. Costs for merged health states are estimated as weighted averages of their constituent costs, with weights defined by the baseline prevalence of the four depression states. The same prevalence was assumed for each cycle as the available prevalence estimate was representative of an average distribution over time. Utilities were mapped from incremental gains in HAMD. These models with ‘fully merged’ states ignore any prior clinical belief that costs or utilities are different between the states (Table 3). Finally, we consider ‘partially merged’ models, where outward transition probabilities across states are assumed to be equal but costs and HAMD, and therefore utilities, associated with the states are assumed to be different.

The HAMD>2 threshold was the most cost effective at a willingness-to-pay threshold of £20,000 for all but the two-state model, where “no treatment at any HAMD threshold” was most cost effective (Table 1). The lower cycle costs for mild depression (£110 treated, £49 untreated) than for depression of any severity in the two-state model (£186 treated, £149 untreated) explain the substantial difference in decision recommendation (Table 2). The EVPI results indicate a short-term trial with a 12-week follow-up is cost effective under all models, though the absolute EVPI estimates vary from approximately £70 to £95 million between the models. A long-term trial, with 2 years of follow-up to better inform the Markov model components, is not likely to be cost effective under any of the models except the two-state model. However, when costs and utilities differ but the outward transition probabilities are merged, the decision and research recommendations are the same across all models (Table 1).

**Table 1:** Results of cost-effectiveness value of information analyses for PANDA based on possible models for depression

Model	Optimal strategy	INB (£) of optimal strategy at willingness to pay £20,000^a^	P(CE) of optimal strategy at willingness to pay £20,000^b^	EVPI (£million)	EVPPI short term (£million)	EVPPI long term (£million)
Four-state (full)	HAMD > 2	223 (−217 to 798)	0.64	80.04	67.29	0
Two-state^c^	No treatment	NA	0.61	95.61	103.62	4.11
Three-state (Mod-Severe)	HAMD > 2	224 (−213 to 805)	0.67	74.88	62.26	0
Three-state (Mild-Mod)	HAMD > 2	234 (−205 to 830)	0.68	70.70	60.53	0
Two-state unconstrained costs^d^	HAMD > 2	225 (−214 to 812)	0.65	77.95	65.45	0
Three-state (Mod-Severe) unconstrained costs	HAMD > 2	224 (−212, 813)	0.65	77.41	64.88	0
Three-state (Mild-Mod) unconstrained costs	HAMD > 2	228 (−205, 830)	0.65	77.06	64.61	0

*CE* cost-effective, *EVPI* expected value of perfect information, *EVPPI* expected value of partial perfect information, *HAMD* Hamilton Depression Rating scale, *INB* incremental net benefit, *Mod-Severe* moderate-severe, *NA*, *PANDA* Prescribing ANtiDepressants that will leAd to a clinical benefit study

^a^Expected INB of treatment if HAMD > 2 strategy vs. no treatment

^b^ *P*(CE) is probability of treatment if HAMD > 2 strategy has highest net benefit

^c^No treatment was the most CE strategy under the two-state model with *P*(CE) = 0.61, treat if HAMD > 25 was second most CE with INB of −2 (−24, 26) and *P*(CE) = 0.32, while HAMD > 2 had an INB of −306 (−757, 289) and *P*(CE) of 0.01 under the two-state model

^d^Unconstrained costs models use four states for costs and HAMD/utilities but merged/constrained models for transition probabilities

**Table 2:**
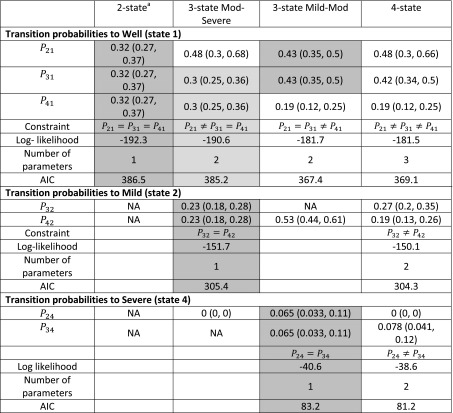

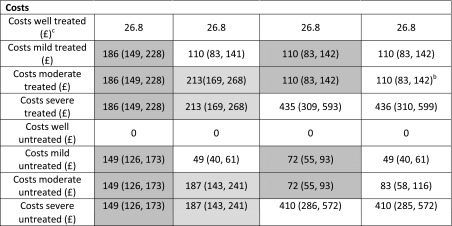
Comparison of transition probabilities and costs for the four Markov cost-effectiveness depression models

For each destination state (well, mild, moderate, severe) the likelihood and AIC are given corresponding to the constraint on the probabilities of transition into this state implied by each model. Models with lower AIC are preferred. Shaded cells indicate parameters that are constrained to be equal in each model

*AIC* Akaike information criterion

*P*_24_ is unconstrained in the Mod-Severe model; *P*_42_ is unconstrained in the Mild-Mod model

^a^Values are mean and 95% credible intervals

^b^Clinical opinion was that costs for mild and moderate treated patients in the four-state model should be the same

^c^Costs for well patients receiving antidepressants is only the cost of the drug, which is fixed by the British National Formulary list price

### Comparison of State Structures Using Constraints

We compare models by constraining parameters in the full (four-state) model to produce models that are equivalent to those with two or three states. We label the four health states as 1 (well), 2 (mild), 3 (moderate) and 4 (severe). The multi-state models being compared are illustrated in Fig. 5.

The four-state model is equivalent to the two-state model if the ‘recovery rates’ are constrained to be independent of depression severity, thus $P_{21} = P_{31} = P_{41}$, and if the costs and HAMD/utilities of the mild, moderate and severe states are assumed to be equal to those of the single depressed state in the two-state model. To constrain the four-state model to be equivalent to the three-state ‘Mod-Severe’ model, we constrain the recovery rates to ‘well’ and the rates to ‘mild’ to be the same, $P_{31} = P_{41}$ and $P_{32} = P_{42}$, respectively, and constrain the costs and HAMD/utilities of the states to be equal. Likewise, the four-state model is constrained to the three-state ‘Mild-Mod’ model by constraining the recovery rates to ‘well’ and the progression rates to ‘severe’, $P_{21} = P_{31}$, $P_{24} = P_{34}$, along with the costs and HAMD/utilities for the mild and moderate states. Other transition probabilities, such as the probabilities of relapse ($P_{12}$, $P_{13} ,P_{14}$), are unaffected by the constraints.

Each constraint is assessed by comparing the likelihood and AIC contributions, describing how well the resulting model fits when estimated using corresponding observed transitions between states. Full details of this method are given in Appendix 3 in ESM. The log-likelihood and AIC for each potential constraint are given in Table 2. An example code to conduct the comparisons in the R statistical software [32] is presented in Appendix 6 in ESM.

Under the unconstrained four-state model, the estimated recovery rates to well are substantially different for a patient with severe depression, thus $P_{41} \ne P_{31}$ and $P_{41} \ne P_{21}$. This is shown formally by the lower AIC for $P_{21} \ne P_{31} \ne P_{41}$ compared with the constraints where $P_{31} = P_{41}$ or $P_{21} = P_{41}$. However, the recovery rates are similar between mild and moderate, thus the AIC is not changed substantially when moving between $P_{21} = P_{31}$ and $P_{21} \ne P_{31}$. The differences between $P_{32}$ and $P_{42}$ and between $P_{24}$ and $P_{34}$ under the four-state model are less striking. This is confirmed by the small difference in AIC between $P_{32} = P_{42}$and $P_{32} \ne P_{42}$, and between $P_{24} = P_{34}$ and $P_{24} \ne P_{34}$. Thus, on the basis of transition probabilities, there is a negligible difference between the three-state Mild-Mod and four-state models, and these are both preferred over the two- and three-state Mod-Severe models, as expected.

Second, we compare the costs informally because these were based on expert belief. The treated costs are the same, though the untreated costs are slightly different, between mild and moderate. Thus, there is some evidence that a model with unconstrained costs is more appropriate. The costs for severe depression are substantially different from mild and moderate depression, arguing against the two-state and ‘Mod-Severe’ models. Prior judgement deemed that utilities are primarily determined by severity of depression, which broadly favours models that have finer classifications of HAMD.

Based on the chosen model, ‘treat if HAMD > 2’ is the optimal strategy. Because the model extrapolates beyond HAMD-D scores included in trials, we conclude that anti-depressant medications are cost effective over the range of HAMD scores included in the trials. We also conclude that there is likely to be value in a short-term trial that recruits patients with milder disease (lower HAMD scores); however, a long-term follow-up is not likely to be cost effective.

## Application to a Model Informed by Published Parameters: CECaT

In this application, there are no individual-level data. Instead, the transition probabilities out of the states being considered for merging are obtained from published estimates. To formally compare the state structures, we have to derive the implicit transition counts underlying the published data.

The Cost-Effectiveness of non-invasive Cardiac Testing (CECaT) study [33] was a randomised trial of diagnostic strategies for CAD, comparing angiography alone with three non-invasive functional tests (followed by confirmatory angiography if positive). Following the trial, a Markov multi-state health economic model was developed, based on previous models by Mowatt et al. [34, 35] and Kuntz et al. [36]. The full structure and assumptions are detailed by Thom [17]. Briefly, a patient with suspected CAD receives one of five alternative diagnostic test strategies and is assigned a diagnosed severity, as a result of which they may receive either medical management or revascularisation. The diagnosed severity may be incorrect because the tests are not perfect and vary in their sensitivity and specificity. The model then proceeds with an annual cycle for 30 years, and at each cycle, a patient may have a myocardial infarction and/or die from any cause.

In these models, CAD severity is categorised into discrete states, representing the increasing risk of myocardial infarction and death, and the increasing need for revascularisation. Mowatt et al. [34, 35] used three risk states: low (no CAD), medium (CAD in one or two vessels excluding the left main stem) or high (CAD in three or more vessels and poor left-ventricular function, or disease in the left main stem). We compare the three-state categorisation with a model where medium- and high-risk states are merged, giving two states representing no CAD or CAD. While the three-state representation is typically used in the literature, it relies on having sufficient information about the differences between medium and high risk to justify separating them. Under the two models, the optimal diagnostic strategy at conventional cost-effectiveness thresholds, and extent of decision uncertainty, are different [17].

Table 3 shows published data used in the full model. The risk of death relative to no CAD differs (significantly) between the two risk groups, but the probability of non-fatal myocardial infarction, the costs and the utilities are similar between the medium- and high-risk groups. The 95% confidence intervals for the state-specific relative risks of death do not overlap, suggesting that they are different enough to merit separation in the model. For a more formal comparison, we derive the implicit data from which these relative risks were obtained: the numbers of people dying in 1 year, and associated denominators, for medium and high risk. Appendix 4 in ESM details how this is done. The problem can then be framed as a comparison of two statistical models for a pair of binomially distributed observations (126 deaths out of 571 in medium risk, and 259 out of 754 in high risk): one model with different probabilities of death, and one where the death probability is constrained to be the same, between medium and high risk. These models have AICs of 17.4 and 39.6, respectively, strongly favouring separate risk states (Table 3).

**Table 3:** Published and derived data on parameters of the CECaT model of coronary artery disease, and AIC difference assessing the constraint that the corresponding parameters are equal between medium- and high-risk states (positive AIC difference favours different parameters)

	Medium risk	High risk	AIC (medium = high) − AIC (medium ≠ high)
Published parameter estimates (with 95% CI)			
Relative risk of death (vs. no CAD)	2.3 (1.9–2.8)	3.6 (3.1–4.1)	
Annual risk of non-fatal MI	0.022 (0.016–0.029)	0.028 (0.021–0.035)	
Derived event count data			
Number/denominator of deaths in 1 year (%)	126/571 (22)	259/754 (34)	22.2
Number/denominator of non-fatal MIs (%)	39/1717 (2.2)	62/2159 (2.8)	−0.6
Summary of individual-level data (mean, SD, sample size)			
Costs	1530, 880, *n* = 59	1930, 1070, *n* = 19	1.4
Utilities	0.81, 0.12, *n* = 59	0.78, 0.21, *n* = 19	−1.4

*AIC* Akaike information criterion, *CAD* coronary artery disease, *CI* confidence interval, *CECaT* Cost Effectiveness of non-invasive Cardiac Testing, *MI* myocardial infarction, *SD* standard deviation

A similar analysis is performed for the risk of non-fatal myocardial infarction, which has overlapping confidence intervals between medium and high risk, though this does not necessarily imply a non-significant difference. An AIC difference of −0.6, however, mildly favours a common risk between the ‘medium’ and ‘high’ states.

The costs and utilities used for the medium- and high-risk states in the economic model (excluding the costs of revascularisation) were estimated from the subset of patients in the CECaT trial whose CAD severity was known. With only 19 of these patients in high risk and 59 in medium risk, it is not clear from the data in Table 3 whether we can assume that expected cost and utility are different between medium and high risk. To assess this formally, generalised linear regression models were fitted to the individual-level cost and utility outcomes by maximum likelihood in R [32], using a gamma distribution for the costs, and a truncated normal distribution for the utilities. The AIC marginally favours a model with different mean costs (AIC difference 1.4) and a model with common mean utility (AIC difference −1.4) between medium and high risk.

Thus, in this model, separating medium- and high-risk states is strongly justified based on their different mortality rates. Though within this structure, there is some evidence that constraining the myocardial infarction rates and utilities to be common between the states will lead to a better trade-off between model fit and model complexity, or bias and precision. Appendix 7 in ESM provides an example R code for all the likelihood and AIC calculations in this example.

## Discussion

Currently, state structure choices are made informally, based upon clinical opinion or availability of data, or compared through simple scenario analyses [2, 7–9]. In this article, we have developed a formal statistical basis to compare state structures in cost-effectiveness models. Specifically, two or more similar states in a transition model can be merged if they have the same consequences for a patient who enters them. The models are then compared by assessing a constraint on these consequences using standard statistical methods, if the parameters are estimated from data, or by expert belief. Thus, we can decide whether the risk of bias in a more parsimonious model outweighs the reduced uncertainty from such a model. While assessing constraints on parameters is common practice, we have shown that models with merged states and models with constrained parameters can be used interchangeably. We proved that this method works for comparing any pair of structures where the states to be merged have the same exit states (Sect. 2.2) and that the method can be adapted to work if they have different exit states (Sect. 2.3). We also showed this method to be valuable even if state structure uncertainties do not affect the current treatment decision as the value of further research, quantified by the EVPI, expected value of partial perfect information or the expected value of sample information, may be sensitive to structural choices [14].

Statistical methods to assess the equality of model parameters require that the data used to estimate those parameters are available, to form the likelihood. For transition probabilities, the number of individuals who are observed to move between each pair of states in a time period, and denominators are required. The Prescribing ANtiDepressants that will leAd to a clinical benefit (PANDA) study used randomised controlled trials but our methods apply to any source, including registries or cohort studies, which provide the data necessary to estimate transition probabilities. We recommend using the data to choose the appropriate state structure before building the full model. Individual patient data were not available for the CECaT model. W recreated the numerators and denominators by assuming that the split between risk groups was the same across randomised arms of the trial, which should approximately hold if randomisation was adequate. To aid such calculations, we recommend that data of this form are published routinely.

Constraints for state selection can also be applied to continuous time multi-state models, which have been advocated for use in health economic modelling [37, 38], as we show in Appendix 5 in ESM, and to the selection of structures for patient-level simulation and heterogeneity models through the inclusion of covariates on the transition probabilities and comparing their effects between states. The principle should also extend to non-Markov multi-state models but this needs to be investigated. In a Bayesian model comparison, expert belief can be used by placing prior probabilities on parameters or model structures and combining with data via Bayes theorem.

Conversely, our method deals only with comparing multi-state structures. Further research into formal statistical methods for other forms of structural uncertainty is required. The choice between continuous and discrete outcome models is difficult. A multi-state model for changes in disease severity is essentially a continuous outcome model, where ranges of the outcome are constrained to have equivalent costs, utilities and future disease progression. However, there is no routinely applicable method to constrain a multi-level regression model, for example, to be equivalent to a multi-state model. Consideration is also required for more complex models, such as dynamic transmission models in infectious diseases [6, 39].

## Conclusion

We have developed a formal method to parameterise state structure uncertainty using constraints on the parameters of the most complex model and have illustrated its wide applicability through examples in depression and CAD. Further research is required for structural uncertainty in non-multi-state cost-effectiveness models.

## Electronic supplementary material

Below is the link to the electronic supplementary material.

Supplementary material 1 (DOCX 72 kb)

## References

[1] Brennan A, Chick SE, Davies R (2006). A taxonomy of model structures for economic evaluation of health technologies. Health Econ.

[2] Briggs AH (2012). Model parameter estimation and uncertainty analysis: a report of the ISPOR-SMDM Modeling Good Research Practices Task Force Working Group-6. Med Decis Making.

[3] National Institute for Health and Care Excellence. Guide to the methods of technology appraisal: process and methods guides. http://publications.nice.org.uk/pmg9. 27905712

[4] Raiffa H, Schlaifer R. Applied statistical decision theory. Wiley Classics Library Ed. New York: Wiley; 2000. p. 356.

[5] Frederix GW (2014). The impact of structural uncertainty on cost-effectiveness models for adjuvant endocrine breast cancer treatments: the need for disease-specific model standardization and improved guidance. Pharmacoeconomics.

[6] Brisson M, Edmunds WJ (2006). Impact of model, methodological, and parameter uncertainty in the economic analysis of vaccination programs. Med Decis Making.

[7] Siebert U (2012). State-transition modelling: a report of the ISPOR-SMDM Modelling Good Research Practices Task Force-3. Value Health..

[8] Jackson CH (2011). A framework for addressing structural uncertainty in decision models. Med Decis Making.

[9] Pitman R (2012). Dynamic transmission modeling: a report of the ISPOR-SMDM Modeling Good Research Practices Task Force-5. Value Health.

[10] Akaike H (1974). A new look at the statistical model identification. IEEE Trans Autom Control.

[11] Jackson CH, Thompson SG, Sharples LD (2009). Accounting for uncertainty in health economic decision models by using model averaging. J R Stat Soc Ser A.

[12] Spiegelhalter DJ (2002). Bayesian measures of model complexity and fit. J R Stat Soc Ser B.

[13] Jackson CH, Sharples LD, Thompson SG (2010). Structural and parameter uncertainty in Bayesian cost-effectiveness models. J R Stat Soc Ser C.

[14] Price M (2011). Model averaging in the presence of structural uncertainty about treatment effects: influence on treatment decision and expected value of information. Value Health.

[15] Thom H (2015). State selection in Markov models for panel data with application to psoriatic arthritis. Stat Med.

[16] Strong M, Oakley JE, Chilcott J (2012). Managing structural uncertainty in health economic decision models: a discrepancy approach. J R Stat Soc Ser C.

[17] Thom H (2013). Structural uncertainty in cost-effectiveness models.

[18] Briggs AH, Ades A, Price M (2003). Probabilistic sensitivity analysis for decision trees with multiple branches: use of the Dirichlet distribution in a Bayesian framework. Med Decis Making.

[19] Kirsch I (2008). Initial severity and antidepressant benefits: a meta-analysis of data submitted to the Food and Drug Administration. PLoS Med.

[20] Fournier JC (2010). Antidepressant drug effects and depression severity. JAMA.

[21] Gibbons RD (2012). Benefits from antidepressants. Arch Gen Psychiatry.

[22] American Pychiatric Assocation (2000). Handbook of pyschiatric measures.

[23] Kessler D (2009). Therapist-delivered internet psychology for depression in primary care: a randomised controlled trial. Lancet.

[24] Kendrick T (2009). Randomised controlled trial to determine the clinical effectiveness and cost-effectiveness of selective serotonin reuptake inhibitors plus supportive care, versus supportive care alone, for mild to moderate depression with somatic symptoms in primary care: the THREAD (THREshold for AntiDepressant response) study. Health Technol Assess.

[25] Chalder M (2012). Facilitated physical activity as a treatment for depressed adults: randomised controlled trial. BMJ.

[26] Curtis L (2014). Unit costs of health and social care 2013.

[27] British national formulary. London: BMG Group and Pharmaceutical Press; 2013.

[28] Henssler J, et al. Long-term acute-phase treatment with antidepressants, 8 weeks and beyond: a systematic review and meta-analysis of randomized, placebo-controlled trials. J Clin Psychiatry. 2017. doi:10.4088/JCP.15r10545**(Epub ahead of print)**. 10.4088/JCP.15r10545 28068463

[29] Kounali D, Lewis G, Ades A. Instrument responsiveness to treatment effects in depression: a meta-analytic approach. Clin Epidemiol. 2016. 10.1016/j.jclinepi.2016.03.005 26994662

[30] Ades AE (2015). Simultaneous synthesis of treatment effects and mapping to a common scale: an alternative to standardisation. Res Synth Methods.

[31] Lu G, Kounali D, Ades AE (2014). Simultaneous multioutcome synthesis and mapping of treatment effects to a common scale. Value Health.

[32] R Core Team. R: a language and environment for statistical computing. Vienna: R Foundation for Statistical Computing; 2015.

[33] Thom H (2014). Cost-effectiveness of initial stress cardiovascular MR, stress SPECT or stress echocardiography as a gate-keeper test, compared with upfront invasive coronary angiography in the investigation and management of patients with stable chest pain: mid-term outcomes from the CECaT randomised controlled trial. BMJ Open.

[34] Mowatt G, et al. Systematic review of the effectiveness and cost-effectiveness, and economic evaluation, or myocardial perfusion scintigraphy for the diagnosis and management of angina and myocardial infarction. Health Technol Assess. 2004;8(30):iii–iv, 1–207. 10.3310/hta8300 15248938

[35] Mowatt G, et al. Systematic review of the clinical effectiveness and cost-effectiveness of 64-slice or higher computed tomography angiography as an alternative to invasive coronary angiography in the estimation of coronary artery disease. Health Technol Assess. 2008;12(17):iii–iv, ix–143. 10.3310/hta12170 18462576

[36] Kuntz KM (1999). Cost-effectiveness of diagnositc strategies for patients with chest pain. Ann Intern Med.

[37] Welton N, Ades A (2005). Estimation of Markov chain transition probabilities and rates from fully and partially observed data: uncertainty propagation, evidence synthesis, and model calibration. Med Decis Making.

[38] Price M, Welton N, Ades A (2011). Parameterization of treatment effects for meta-analysis in multi-state Markov models. Stat Med.

[39] Bilcke J (2011). Accounting for methodological, structural, and parameter uncertainty in decision-analytic models: a practical guide. Med Decis Making.

[40] Yusuf S (1994). Effect of coronary artery bypass graft surgery on survival: overview of 10-year results from randomised trials by the Coronary Artery Bypass Graft Surgery Trialists Collaboration. Lancet.

[41] Norris JR. Markov chains. 1st ed. Cambridge series on statistical and probabilistic mathematics. Cambridge: Cambridge University Press; 1998. p. 237.

[42] Cox DR, Miller HD (1965). The theory of stochastic processes.

[43] Lunn D, et al. The BUGS book: a practical introduction to Bayesian analysis. Texts in statistical science. Boca Raton: CRC Press; 2013. p. 381.

