# Supplementary material for: Using Parameter Constraints to Choose State Structures in Cost-Effectiveness Modelling
Source: Pharmacoeconomics. 2017 Mar 24;35(9):951–62. doi: 10.1007/s40273-017-0501-9 (PMC5563360; doi:10.1007/s40273-017-0501-9)
Supplement: Supplementary file 1 — Supplementary material 1 (DOCX 72 kb) [file 40273_2017_501_MOESM1_ESM.docx]

## Appendix 1: Proof constrained split state model gives same predictions as merged state model

The expected survival in the merged model is given by the recursive equation

$$E\left( S_{M} \right)=P_{D}\times1+\left( 1-P_{D} \right)\times(1+E\left( S_{M} \right))$$

Where $P_{D}$ is the probability of death in each cycle for patients in merged low and high severity CAD states. The expected survival time survival is therefore $E\left( S_{M} \right)=\frac{1}{P_{D}}$. Similarly, in the split model the expected survival time from entry into the high severity state is $E\left( S_{H} \right)=\frac{1}{P_{HD}}$. The expected time to survival for low severity individuals is given by

$$E\left( S_{L} \right)=P_{LD}+P_{LH}\left( 1+E\left( S_{H} \right) \right)+(1-P_{LD}-P_{LH})(1+E\left( S_{L} \right))$$

where an extra cycle is included in the second term for patients transiting to death by first passing through high severity CAD. Solving gives $E\left( S_{L} \right)=\frac{1}{P_{LD}}$. Thus if we constrain $P_{LD}=P_{HD}=P_{D}$ the models will give the same expected survival. We now prove that under the constraint, the split model will give the same estimate of $P_{D}$ as the merged model.

To prove this, first denote the number of patients initially in the high and low severity CAD states as $n_{H}$ and $n_{L}$, respectively. Also denote the number of patients transiting from high severity CAD to Death as $r_{HD}$, from low severity to Death as $r_{LD}$ and from low to high as $r_{LH}$. As explained in the literature[18], the likelihood over the observed transitions for the merged state model can be written as

$L_{merged}\left( P_{D} \right)\propto P_{D}^{r_{LD}+r_{HD}}\times{(1-P_{D})}^{n_{L}+n_{H}-r_{HD}-r_{LD}}$ (2)

And the likelihood for the split state model can be written as

$L_{split}\left( P_{LH},P_{HD},P_{LD} \right)\propto P_{LH}^{r_{LH}}P_{LD}^{r_{LD}}P_{HD}^{r_{HD}}{(1-P_{LH}-P_{LD})}^{n_{L}-r_{LH}-r_{LD}}{(1-P_{HD})}^{n_{H}-r_{HD}}$ (3)

In order to show that the likelihood of the split state model is in fact proportional to that of the merged state model, we introduce the parameterization

$P_{LH}=\rho(1-P_{LD})$ (4)

This replaces the nuisance parameter $P_{LH}$ with the parameter $\rho$, the proportion of patients remaining alive who transit to high severity CAD, which can be expressed

$$\rho=\frac{P_{LH}}{(1-P_{LD})}$$

The likelihood of the split state model in Equation (3) then becomes

$L_{split}\left( P_{HD},P_{LD} \right)\propto{(1-P_{LD})}^{r_{LH}}P_{LD}^{r_{LD}}{(1-P_{LD})}^{n_{L}-r_{LH}-r_{LD}}P_{HD}^{r_{HD}}{(1-P_{HD})}^{n_{H}-r_{HD}}$ (5)

where the proportionality factor includes terms in $\rho$. If we now impose the constraint (1) that $P_{HD}=P_{LD}=P_{D},$the likelihood of the split state model becomes

$L_{Constrained}(\rho,P_{D})\propto P_{D}^{r_{LD}+r_{HD}}{(1-P_{D})}^{r_{LH}+n_{L}-r_{LH}-r_{LD}+n_{H}-r_{HD}}$ (6)

Which, after rearranging and cancelling, is seen to be proportional to the likelihood of the merged model in Equation (2). Maximising the constrained likelihood in Equation (6) and the likelihood for the merged model will give the same estimate of the $P_{D}$ parameter so the models will give the same predictions. Likewise, in a Bayesian setting, if the same prior on $P_{D}$ is used, the posterior distributions will be the same.

## Appendix 2: General proof for merging n states with m exits

We aim to show that the m+n state model illustrated in Figure 3 can be constrained so as to give equivalent predictions to a model with only m+1 states. The states to be merged are $A_{1},\ldots,A_{n}$ and their exit states are $E_{1},\ldots,E_{m}$. Transition probabilities are labelled $P_{A_{j}E_{i}}$ and $P_{A_{j}A_{k}}$ for transitions from merging states $A_{j}$ to exit state $E_{i}$ and between the merging states $A_{j}$ and $A_{k}$, respectively. Transition probabilities $P_{E_{k}E_{i}}$ between exit states are not affected by merging and are not of interest. The probabilities of transiting from the exit states to the merging states are unaffected by the merging as the sum

$$\sum_{j=1}^{n} P_{{E_{i}A}_{j}}$$

is unchanged and the precise assignment of transitions among the n merging states is irrelevant.

The necessary constraints are

$$P_{A_{j}E_{i}}=P_{i}, \forall j,i.$$

For each of the states $A_{j}$ there are $n-1$ nuisance parameters; each of these is labelled $\rho_{jk}$ and is associated with a transition to another merged state $A_{k}$, with $k\neq j$. These re-parameterisations, for $j\in1,\ldots,n$, are

$P_{A_{j}A_{1}}=\rho_{j1}\left( 1-\sum_{i=1}^{m} P_{A_{j}E_{i}} \right)$ for $j\neq1$

So $\rho_{j1}$ is the proportion of the patients who do not transit from $A_{j}$ to any exit state who transit to state $A_{1}$

$\rho_{j1}=\frac{P_{A_{j}A_{1}}}{\left( 1-\sum_{i=1}^{m} P_{A_{j}E_{i}} \right)}$ for $j\neq1$

A similar $\rho_{jk}$ is introduced for every $k\neq j$ representing the proportion of patients who do not transit from $A_{j}$ to any exit state or states $A_{1},\ldots,A_{k-1}$ but who do transit to state $A_{k}$:

$$\rho_{jk}=\frac{P_{A_{j}A_{k}}}{\left( \prod_{l\neq j,l<k} \left( 1-\rho_{jl} \right) \right)\left( 1-\sum_{i=1}^{m} P_{A_{j}E_{i}} \right)}$$

So

$$P_{A_{j}A_{k}}=\rho_{jk}\left( \prod_{l\neq j,l<k} \left( 1-\rho_{jl} \right) \right)\left( 1-\sum_{i=1}^{m} P_{A_{j}E_{i}} \right)$$

This is repeated for every state up to n. The essential feature is that every probability satisfies

$$P_{A_{j}A_{k}}\propto\left( 1-\sum_{i=1}^{m} P_{A_{j}E_{i}} \right)$$

where the factor of proportionality is dependent only on the nuisance parameters $\rho_{jk}$. Under the constraint, these are further simplified to

$$P_{A_{j}A_{k}}\propto\left( 1-\sum_{i=1}^{m} P_{i} \right).$$

The components of the likelihood that depend on the parameters common to the split $m+n$ state model and the merged $m+1$ model are

$$L_{split}(P_{A_{j}E_{i}},P_{A_{j}A_{k}})$$

$$\propto\prod_{j=1}^{n} \left[ \left( \prod_{k\neq j} P_{A_{j}A_{k}}^{r_{A_{j}A_{k}}} \right) \left( \prod_{i=1}^{m} P_{A_{j}E_{i}}^{r_{A_{j}E_{i}}} \right)\left( 1-\sum_{k\neq j} P_{A_{j}A_{k}}-\sum_{i=1}^{m} P_{A_{j}E_{i}} \right)^{n_{A_{j}}-\sum_{k\neq j} r_{A_{j}A_{k}}-\sum_{i=1}^{m} r_{A_{j}E_{i}}} \right]$$

Under the constraint this becomes

$$\propto\prod_{j=1}^{n} \left[ \left( \prod_{k\neq j} P_{A_{j}A_{k}}^{r_{A_{j}A_{k}}} \right) \left( \prod_{i=1}^{m} P_{i}^{r_{A_{j}E_{i}}} \right)\left( 1-\sum_{k\neq j} P_{A_{j}A_{k}}-\sum_{i=1}^{m} P_{i} \right)^{n_{A_{j}}-\sum_{k\neq j} r_{A_{j}A_{k}}-\sum_{i=1}^{m} r_{A_{j}E_{i}}} \right]$$

Under re-parameterisation, and dropping terms in $\rho_{jk}$, this becomes

$$\propto\prod_{j=1}^{n} \left[ \left( 1-\sum_{i=1}^{m} P_{i} \right)^{\sum_{k\neq j} r_{A_{j}A_{k}}} \left( \prod_{i=1}^{m} P_{i}^{r_{A_{j}E_{i}}} \right)\left( 1-\sum_{i=1}^{m} P_{i} \right)^{n_{A_{j}}-\sum_{k\neq j} r_{A_{j}A_{k}}-\sum_{i=1}^{m} r_{A_{j}E_{i}}} \right].$$

Grouping and cancelling terms this becomes

$$=\prod_{j=1}^{n} \left[ \left( \prod_{i=1}^{m} P_{i}^{r_{A_{j}E_{i}}} \right)\left( 1-\sum_{i=1}^{m} P_{i} \right)^{n_{A_{j}}-\sum_{i=1}^{m} r_{A_{j}E_{i}}} \right].$$

This is proportional to the likelihood for an $m+1$ state model and will thus give the same maximum likelihood or Bayesian estimates for the transition probabilities $P_{i}$from the merged state to each exit state. The remaining transition probabilities between the exit states ($P_{E_{k}E_{i}}$) remain unchanged and the nuisance parameters $\rho_{jk}$ will be as in the unconstrained $m+n$ state model. This is the required result.

## Appendix 3. Assessing constraints on transition probabilities using likelihoods

Consider a general *m*-state discrete-time Markov model with transition probabilities $\left\{ P_{rs}:r, s=1,\ldots,m \right\}$, $\sum_{s=1}^{m} P_{rs}=1$ for each $r$*,* and data giving the number of transitions $n_{rs}$ between each state $r,s=1, \ldots, m$ over a single cycle, and denominators $n_{r}=\sum_{s} n_{rs}$. The overall likelihood is

$$L\left( \left\{ P_{rs}:r, s=1,\ldots m \right\} \right| \left\{ n_{rs}:r,s=1, \ldots, m \right\})=\prod_{r, s} {P_{rs}}^{n_{rs}}$$

and the corresponding maximum likelihood estimates are $\hat{P_{rs}}=n_{rs}/n_{r}$.

In the PANDA application (Section 3), for example, there are $m=4$ states, and the likelihood relating to the merged IPCRESS, THREAD, and TREAD transition count data is

$$L\left( \left\{ P_{rs}:r=2,3,4, s=1,2,3,4 \right\} \right| \left\{ n_{rs}:r=2,3,4, s=1,2,3,4 \right\})=\prod_{r, s} {P_{rs}}^{n_{rs}}$$

The transitions from state $r=1$ are unaffected by any of our model choices, and estimated from an independent set of data, thus the corresponding terms are omitted from the likelihood in this illustration.

The choice of whether to merge some or all of a set of states ($r=2, 3,$and $4$ say, as in the PANDA example) involves assessing whether some or all of the transition probabilities $P_{2s}$, $P_{3s}$ and $P_{4s}$ can be assumed to be equal. This should be done for each potential destination state $s$ to which transitions from 2, 3 and 4 can occur. For example, without loss of generality, take$s=1$; In PANDA, this is the “well” state. By reparameterising as in Appendix 1-2, it can be shown that the full likelihood is proportional to

$${P_{21}}^{n_{21}}{(1-P_{21})}^{n_{2}{-n}_{21}}{P_{31}}^{n_{31}}{(1-P_{31})}^{n_{3}-n_{31}}{P_{41}}^{n_{41}}{(1-P_{41})}^{n_{4}-n_{41}}$$

where the constant of proportionality does not involve the parameters of interest, here $P_{21}$, $P_{31}$, $P_{41}$. The likelihood is thus a product of terms which change according to the constraint on the transition probabilities implied by the model, and other terms which are equal between models and can be ignored.

Hence the maximum likelihood estimates $\hat{P_{21}}$, $\hat{P_{31}}$, $\hat{P_{41}}$ under each assumption can then easily be derived as, e.g. $\hat{P_{21}}=n_{21}/n_{2}$, $\hat{P_{31}}=n_{31}/n_{3}$, $\hat{P_{41}}=n_{41}/n_{4}$, with no constraint, $\hat{P_{21}}={\hat{P_{31}}=(n}_{21}+ n_{31})/{(n}_{2}+n_{3})$ under $P_{21}=P_{31}$, and $\hat{P_{21}}={\hat{P_{31}}=\hat{P_{41}}=(n}_{21}+ n_{31}+ n_{41})/{(n}_{2}+n_{3}+n_{4})$ if all three probabilities are assumed equal.

The AIC for comparing models then follows from the maximised likelihood:

$$AIC= -2 log L(\hat{P_{21}},\hat{P_{31}},\hat{P_{41}})+2d$$

where $d$ is the number (dimension) of parameters being estimated, for example here this is $d=3$ with no constraint, $d=2$ if $P_{21}=P_{31}\neq P_{41}$, and $d=1$ if $P_{21}=P_{31}=P_{41}$. Models with lower AIC are estimated to have better predictive ability, equivalently, a better compromise between potential bias and precision of their estimates.

A similar comparison of likelihoods and AIC can be made for other destination states $s$. In the PANDA model, for example, a further comparison is made to assess whether $P_{24}=P_{34},$ by expressing the likelihood as proportional to

$${P_{24}}^{n_{24}}{(1-P_{24})}^{n_{2}{-n}_{24}}{P_{34}}^{n_{34}}{(1-P_{34})}^{n_{3}-n_{34}}$$

and a further comparison to assess whether $P_{32}=P_{42}$, by writing the likelihood as proportional to

$${P_{32}}^{n_{32}}{(1-P_{32})}^{n_{3}-n_{32}}{P_{42}}^{n_{42}}{(1-P_{42})}^{n_{4}-n_{42}}$$

## Appendix 4: Deriving implicit binomial mortality data and AICs for the CECaT model

To represent the effect of CAD on mortality in the absence of revascularisation, the CECaT model uses two published relative risks: 2.3 (95% CI 1.9, 2.8) representing the relative risk of 1-year mortality between medium risk CAD and low risk, and 3.6 (3.1, 4.1) between high risk and low risk, derived by Kuntz et al. from a systematic review of trials comparing revascularisation (by coronary artery bypass graft) with medical management [36, 40]. From these numbers, we derive the implicit binomially-distributed outcomes *b* and *c*, with respective denominators *M* and *H*, representing the numbers of medium and high-risk patients with medically-managed CAD dying in one year. These data can be tabulated as

|  | Dead | Alive | Total |
| --- | --- | --- | --- |
| Low risk | a | L-a | L |
| Medium risk | b | M-b | M |
| High risk | c | H-c | H |

The relative risks are *(b/ M) / (a/L)* = 2.3 and *(c/H) / (a/L)* = 3.6. We also assume the confidence intervals came from the usual approximate standard errors $\sigma_{M},\sigma_{H}$ of the log relative risks, giving

$\sigma_{M}= \sqrt{\frac{1}{a}+\frac{1}{b}-\frac{1}{L}-\frac{1}{M}}$ = $(log \left( 2.3 \right)-\log\left( 1.9 \right))/1.96$

$\sigma_{H}= \sqrt{\frac{1}{a}+\frac{1}{c}-\frac{1}{L}-\frac{1}{H}}$ = $(log \left( 3.6 \right)-\log\left( 3.1 \right))/1.96$

*M and H* can be approximately derived from the report by Yusuf et al. (1994)[40], which stated that 1130 and 1491 patients were in the medium and high risk states respectively at baseline, and 1325 of these were randomised to medical management. Assuming the risk proportions are the same between randomised groups, the number of medically-managed patients in the medium risk category would be *M* = 1325 x 1130 / (1130 + 1491) = 571. Similarly, *H* = 1325 x 1130 / (1130 + 1491) = 754.

Combining the two pairs of equations above to cancel the terms in *a* and *L* gives

$\frac{bH}{cM}=2.3/3.6$ , $\sigma_{M}^{2}- \sigma_{H}^{2}=\frac{1}{b}-\frac{1}{c}-\frac{1}{M}+\frac{1}{H}$

Since we know M, H, $\sigma_{M}^{2}$ and $\sigma_{H}^{2}$ , we can solve these jointly to give b = 126 and c = 259.

The annual risk of non-fatal myocardial infarction was presented by Kuntz et al. as 0.022 (0.016 – 0.029) in the medium risk state and 0.028 (0.021– 0.035) in high risk[36]. These are converted to binomially-distributed data by assuming the point and interval estimates came from quantiles of a Beta(y+1, n-y+1) distribution, which is the posterior under a uniform prior and a binomial observation of y events from a denominator of n.

Thus the likelihood for the probabilities of death in medium and high risk states, given these data, is constructed from the binomial distribution assumed for$b$ and $c$:

$$L\left( p_{M},p_{H} | b,c,M,H \right)={p_{M}}^{b}{(1- p_{M})}^{M-b}{p_{H}}^{c}{(1- p_{H})}^{H-c}$$

The likelihood is maximised under two alternative assumptions: a) $p_{M}\neq p_{H}$ and b) $p_{M}=p_{H}$, giving maximum likelihood estimates $\hat{p_{M}}$ and $\hat{p_{H}}$. This leads to the Akaike Information Criterion (AIC)

$$-2\log(L\left( \hat{p_{M}},\hat{p_{H}} | b,c,M,H \right))+2d$$

where $d$ is the number of parameters that are estimated: 2 under assumption a) and 1 under b). A lower AIC means the corresponding assumption is favoured. Appendix 6 provides R code to calculate these quantities for the probabilities of death and myocardial infarction, and a similar method for comparing the mean cost and utility between the medium and high risk states using the likelihoods of Gamma and truncated normal distributions fitted to individual-level cost and utility data.

## Appendix 5: Proof that constrained split state model gives same predictions as merged state model in continuous time

The choice between continuous time split state and merged state models is illustrated in Figure 6.

#### Figure 6. Comparison of split and merged severity state CAD models in continuous time^a^

^a^$\lambda_{XY}$ is the transition rate between state X to state Y.

The rate of transiting from low to high severity CAD is $\lambda_{LH}$, from low severity CAD to death is $\lambda_{LD}$, from high severity CAD to death is $\lambda_{HD}$, from combined CAD to death is $\lambda_{D}$, while the data $n_{H}$, $n_{L}$, $r_{HD}$, $r_{LD}$, and $r_{LH}$ are as before. Using the Kolmogorov forward equations for continuous-time Markov processes[41, 42] the probability after time t of remaining in the low severity CAD state is

$$P_{LL}\left( t \right)=e^{-\left( \lambda_{LH}+\lambda_{LD} \right)t}$$

And of transiting from low severity CAD to high severity CAD is

$$P_{LH}\left( t \right)=\frac{\lambda_{LH}e^{-\lambda_{HD}t}(1-e^{-\left( \lambda_{LH}+\lambda_{LD}-\lambda_{HD} \right)t})}{(\lambda_{LH}+\lambda_{LD}-\lambda_{HD})}$$

of transiting from low severity CAD to death is

$$P_{LD}\left( t \right)=1-P_{LL}\left( t \right)-P_{LH}(t)$$

of transiting from high severity CAD to death is

$$P_{HD}\left( t \right)=1-e^{-\lambda_{HD}t}$$

and of remaining at high severity CAD is

$P_{HH}\left( t \right)=e^{-\lambda_{HD}t}$.

Applying the constraint

$$\lambda_{LD}=\lambda_{HD}=\lambda_{D}$$

makes the split state model equivalent to the merged state model. For convenience we set $t=1$. The probabilities become

$$P_{LL}\left( t \right)=e^{-\left( \lambda_{LH}+\lambda_{D} \right)}$$

$$P_{LH}\left( t \right)=e^{-\lambda_{D}}-e^{-\left( \lambda_{LH}+\lambda_{D} \right)}$$

$$P_{LD}\left( t \right)=1-e^{-\lambda_{D}}$$

$$P_{HD}\left( t \right)=1-e^{-\lambda_{D}}$$

$$P_{HH}\left( t \right)=e^{-\lambda_{D}}$$

The likelihood of the constrained split state model is therefore proportional to

$$e^{-(\lambda_{LH}+\lambda_{D})\times(n_{L}-r_{LH}-r_{LD})}\times\left( e^{-\lambda_{D}}(1-e^{-\lambda_{LH}}) \right)^{r_{LH}}\times{(1-e^{{-\lambda}_{D}})}^{r_{LD}}\times{(1-e^{-\lambda_{D}})}^{r_{HD}}\times e^{-\lambda_{D}(n_{H}-r_{HD})}$$

After rearranging, this is proportional to

$$e^{-\lambda_{D}(n_{L}+n_{H}-r_{LD}-r_{HD})}\times{(1-e^{-\lambda_{D}})}^{(r_{LD}+r_{HD})}$$

which is the likelihood of the merged state model. Maximising this likelihood, or Bayesian inference under the same prior, therefore gives the same parameter estimates, and the same predictions, as the merged state model.

## Appendix 6: R code to compare 2-state and 4-state depression models using AIC

In this appendix we provide example code to compare the constraints on the 4-state transition probabilities in the depression model. The data (r=transitions, n=number of patients at risk of transition) are included so this example can be used immediately; these are the summaries of transitions from IPCRESS, THREAD, and TREAD that are necessary to reproduce our results. Note that Bayesian estimates of the transition probabilities (calculated using the OpenBUGS software [43]) were used for the economic model since they give a better quantification of uncertainty than the maximum likelihood estimates in the presence of small counts [18]. Although the deviance information criterion (DIC) is more suitable in general for comparing Bayesian models, the models are compared using AIC in this case for simplicity of illustration. AIC gives similar model preferences to DIC in this case, since uniform priors were used.

# Code to evaluate constraints on 4-state transition probabilities for the PANDA model

*# Observed data*

*n2=27; n3=141; n4=139*

*r21=13; r23=0; r24=0*

*r31=59; r32=39; r34=11*

*r41=26; r42=26; r43=47*

*# 4-state minus log likelihood to assess merging moderate and severe states (P_32=P_42)*

*# Unconstrained*

*# P[1] is P_32*

*# P[2] is P_42*

*lh.modsevere.f<-function(P)*

*{*

*return(-r32*log(P[1])-(n3-r32)*log(1-P[1])-*

*r42*log(P[2])-(n4-r42)*log(1-P[2]))*

*}*

*# Constrained P_32=P_42*

*lh.modsevere.e<-function(P)*

*{*

*return(-r32*log(P[1])-(n3-r32)*log(1-P[1])-*

*r42*log(P[1])-(n4-r42)*log(1-P[1]))*

*}*

*# 4-state minus log likelihood to assess merging mild and moderate states*

*# Unconstrained*

*# P[1] is P_24*

*# P[2] is P_34*

*lh.mildmod.h<-function(P)*

*{*

*return(-r24*log(P[1])-(n2-r24)*log(1-P[1])-*

*r34*log(P[2])-(n3-r34)*log(1-P[2]))*

*}*

*# Constrained P_24=P_34*

*lh.mildmod.g<-function(P)*

*{*

*return(-r24*log(P[1])-(n2-r24)*log(1-P[1])-*

*r34*log(P[1])-(n3-r34)*log(1-P[1]))*

*}*

*# 4-state minus log likelihood to assess constraints on P_21, P_31, P_41 (recovery probability)*

*# Unconstrained (b)*

*# P[1] is P_21*

*# P[2] is P_31*

*# P[3] is P_41*

*lh.recovery.b<-function(P)*

*{*

*return(-r21*log(P[1])-(n2-r21)*log(1-P[1])-*

*r31*log(P[2])-(n3-r31)*log(1-P[2])-*

*r41*log(P[3])-(n4-r41)*log(1-P[3]))*

*}*

*# Constrained (a) P_21=P_31=P_41*

*lh.recovery.a<-function(P)*

*{*

*return(-r21*log(P[1])-(n2-r21)*log(1-P[1])-*

*r31*log(P[1])-(n3-r31)*log(1-P[1])-*

*r41*log(P[1])-(n4-r41)*log(1-P[1]))*

*}*

*# Constrained (c) P_21=P_31!=P_41*

*lh.recovery.c<-function(P)*

*{*

*return(-r21*log(P[1])-(n2-r21)*log(1-P[1])-*

*r31*log(P[1])-(n3-r31)*log(1-P[1])-*

*r41*log(P[2])-(n4-r41)*log(1-P[2]))*

*}*

*# Constrained (d) P_21!=P_31=P_41*

*lh.recovery.d<-function(P)*

*{*

*return(-r21*log(P[1])-(n2-r21)*log(1-P[1])-*

*r31*log(P[2])-(n3-r31)*log(1-P[2])-*

*r41*log(P[2])-(n4-r41)*log(1-P[2]))*

*}*

*# Maximum likelihood estimators for the likelihoods*

*mle.modsevere.f<-c(r32/n3,r42/n4)*

*mle.modsevere.e<-(r32+r42)/(n3+n4)*

*mle.mildmod.h<-c(r24/n2+0.001,r34/n3)*

*mle.mildmod.g<-(r24+r34)/(n2+n3)*

*mle.recovery.a<-(r21+r31+r41)/(n2+n3+n4)*

*mle.recovery.b<-c(r21/n2,r31/n3,r41/n4)*

*mle.recovery.c<-c((r21+r31)/(n2+n3),r41/n4)*

*mle.recovery.d<-c(r21/n2,(r31+r41)/(n3+n4))*

*# Calculate the AIC to evaluate the 8 constraints*

*AIC.recovery.a<-2*lh.recovery.a(mle.recovery.a)+2*length(mle.recovery.a)*

*AIC.recovery.b<-2*lh.recovery.b(mle.recovery.b)+2*length(mle.recovery.b)*

*AIC.recovery.c<-2*lh.recovery.c(mle.recovery.c)+2*length(mle.recovery.c)*

*AIC.recovery.d<-2*lh.recovery.d(mle.recovery.d)+2*length(mle.recovery.d)*

*AIC.modsevere.e<-2*lh.modsevere.e(mle.modsevere.e)+2*length(mle.modsevere.e)*

*AIC.modsevere.f<-2*lh.modsevere.f(mle.modsevere.f)+2*length(mle.modsevere.f)*

*AIC.mildmod.g<-2*lh.mildmod.g(mle.mildmod.g)+2*length(mle.mildmod.g)*

*AIC.mildmod.h<-2*lh.mildmod.h(mle.mildmod.h)+2*length(mle.mildmod.h)*

## Appendix 7: R code for the CECaT example (Section 4)

*## Number and denominator of deaths in medium risk*

*dmr <- 126*

*mr <- 571*

*## Number and denominator of deaths in high risk*

*dhr <- 259*

*hr <- 754*

*## Same numbers for myocardial infarction*

*# dmr <- 39; mr <- 1717; dhr <- 62; hr <- 2159*

*dat <- data.frame(risk=c(0,1))*

*dat$out <- cbind(dead=c(dmr,dhr), alive=c(mr-dmr,hr-dhr))*

*## Maximise the binomial likelihoods for these data under two assumptions*

*## a) mortality depends on risk state*

*ress <- glm(out ~ risk, data=dat, family="binomial")*

*## b) mortality common between risk state*

*resm <- glm(out ~ 1, data=dat, family="binomial")*

*AIC(resm)*

*AIC(ress)*

*## AIC difference: positive favours model where mortality depends on risk state*

*AIC(resm) - AIC(ress)*

*## dat is a data frame with variables*

*## "cost" (individual level cost observations)*

*## "util" (individual level utility observations)*

*## "state" (state corresponding to each observation)*

*## Compare costs between medium and high risk states*

*## Assume individual data are drawn from Gamma distribution:*

*## a) model where gamma mean depends on state*

*ress <- glm(cost ~ state, data=dat, family=Gamma)*

*## b) model where gamma mean independent of state*

*resm <- glm(cost ~ 1, data=dat, family=Gamma)*

*AIC(resm) - AIC(ress)*

*### Compare utilities between medium and high risk states*

*## Assume they are drawn from a truncated normal model with truncation points -0.594, 1*

*## Estimate the mean and SD before truncation by maximising the likelihood*

*library(msm)*

*## a) model where mean depends on state*

*minusloglik.split <- function(par){*

*-sum(dtnorm(dat$util[dat$state=="med"], mean=par[1], sd=par[3], lower=-0.594, upper=1, log=TRUE)) -*

*sum(dtnorm(dat$util[dat$state=="hi"], mean=par[2], sd=par[3], lower=-0.594, upper=1, log=TRUE))*

*}*

*## b) model where mean independent of state*

*minusloglik.common <- function(par){*

*-sum(dtnorm(dat$util, mean=par[1], sd=par[2], lower=-0.594, upper=1, log=TRUE))*

*}*

*opt1 <- optim(c(mean(dat$util), sd(dat$util)), minusloglik.common)*

*opt2 <- optim(c(rep(mean(dat$util),2), sd(dat$util)), minusloglik.split)*

*(aic1 <- 2*opt1$value + 2*2) # common: 2 parameters*

*(aic2 <- 2*opt2$value + 2*3) # split: 3 parameters*

*aic1 - aic2*
